# Performance of complex snow cover descriptions in a distributed hydrological model system: A case study for the high Alpine terrain of the Berchtesgaden Alps

**DOI:** 10.1002/wrcr.20219

**Published:** 2013-05-28

**Authors:** M Warscher, U Strasser, G Kraller, T Marke, H Franz, H Kunstmann

**Affiliations:** 1 Institute of Meteorology and Climate Research (IMK-IFU), Karlsruhe Institute of Technology (KIT)Garmisch-Partenkirchen, Germany; 2 Institute of Geography, University of InnsbruckInnsbruck, Austria; 3 Berchtesgaden National Park AdministrationBerchtesgaden, Germany; 4 Institute for Geography, University of AugsburgAugsburg, Germany

## Abstract

[1] Runoff generation in Alpine regions is typically affected by snow processes. Snow accumulation, storage, redistribution, and ablation control the availability of water. In this study, several robust parameterizations describing snow processes in Alpine environments were implemented in a fully distributed, physically based hydrological model. Snow cover development is simulated using different methods from a simple temperature index approach, followed by an energy balance scheme, to additionally accounting for gravitational and wind-driven lateral snow redistribution. Test site for the study is the *Berchtesgaden National Park* (Bavarian Alps, Germany) which is characterized by extreme topography and climate conditions. The performance of the model system in reproducing snow cover dynamics and resulting discharge generation is analyzed and validated via measurements of snow water equivalent and snow depth, satellite-based remote sensing data, and runoff gauge data. Model efficiency (the Nash-Sutcliffe coefficient) for simulated runoff increases from 0.57 to 0.68 in a high Alpine headwater catchment and from 0.62 to 0.64 in total with increasing snow model complexity. In particular, the results show that the introduction of the energy balance scheme reproduces daily fluctuations in the snowmelt rates that trace down to the channel stream. These daily cycles measured in snowmelt and resulting runoff rates could not be reproduced by using the temperature index approach. In addition, accounting for lateral snow transport changes the seasonal distribution of modeled snowmelt amounts, which leads to a higher accuracy in modeling runoff characteristics.

## 1. Introduction

[2] Runoff in Alpine regions is largely controlled by snow accumulation, storage, redistribution, and melting. Generally, the full complexity of the water balance in Alpine regions is only partially understood. High altitudinal gradients, a strong variability of meteorological variables in time and space, unquantified snow cover dynamics, complex and often unknown hydrogeological settings, and heterogeneous land use and soil formations result in high uncertainties in the quantification of the water balance and the prediction of discharge rates [*Klemes*, 1990].

[3] Numerous model approaches exist to simulate snow processes on different scales. Depending on the purpose of the model application, e.g., runoff simulations, flood or avalanche forecasting, glacier mass balance, or small-scale snow physics studies, many snow models have been developed. The approaches can be classified in three different groups with numerous transitions in between (1) index models (e.g., temperature, wind, or radiation index), (2) models that determine the energy balance of a snow pack or surface, and (3) multilayer schemes that additionally simulate processes within the snow pack, e.g., stratification, metamorphism, and the accompanying energy and mass fluxes. Besides, there are formulations to account for additional processes like the interaction between snow and vegetation or for lateral snow transport. An overview of distributed snow modeling is given by *Kirnbauer et al*. [1994] and *Fierz et al*. [2003], of snow process modeling in different applications by *Marsh* [1999], and of snow process integration in land surface models by *Pomeroy et al*. [1998].

[4] In distributed hydrological models, index approaches are most commonly used because of their simplicity, robustness, and efficiency. It is obvious, however, that they are not suitable tools to produce reasonable results for snow cover development in complex Alpine terrain on regional to local scales. Moreover, it is questionable to use temperature index methods in climate change impact assessment studies because of their high sensitivity to temperature change and their inability to adapt to changing systems. This holds particularly in high mountain regions with complex topography that are sensitive to climate change and at the same time, subject to high uncertainties in climate projections [*Smiatek et al*., 2009,^2011^; *Laux et al*., 2011; *Kobierska et al*., 2012].

[5] *Marks et al*. [1999], *Susong et al*. [1999], *Garen and Marks* [2005], *Lehning et al*. [2006], *Strasser* [2008], and *Liston and Elder* [2006] present the application of distributed snow model systems that are suited for mountainous terrain and based on physical descriptions. *Bavay et al*. [2009] and *Kobierska et al*. [2012] use the model developed by *Lehning et al*. [2006] for future runoff simulations. Runoff formation is assessed by a conceptual approach in this case. *Rigon et al*. [2006] recently developed a distributed hydrological model system to simulate coupled mass and energy fluxes in Alpine catchments. They all show—with some limitations—successful applications of distributed snow models to Alpine catchments with high model resolution in time and space. To our knowledge, sophisticated snow modeling methods including lateral snow transport processes have not yet been used and studied in a physically based, fully distributed hydrological model on a regional scale. This study aims at establishing a distributed runoff model of this type that is, in addition, capable of performing scenario runs in the region. This paper will report about the investigation of the influence of different snow model approaches on modeling runoff dynamics in complex, high Alpine terrain.

## 2. Study Area

[6] The investigated catchment of the *Berchtesgadener Ache* (Bavarian Alps, Germany) comprises an area of 432 km^2^. It is characterized by an extreme topography with mountain ranges covering an altitude from 603 to 2713 m above mean sea level (MSL). About one quarter of the catchment area has slopes steeper than 35°. The *Lake Königssee* (603 m MSL) is situated next to the highest and best known summit of the region, the *Watzmann Mittelspitze* (2713 m MSL). The large altitudinal gradient (2110 m) between these two tourist attractions and the resulting different climatic conditions at a horizontal distance of about 3500 m illustrate the large spatial heterogeneity of the catchment. The mean annual precipitation ranges from 1500 mm in the valleys up to 2600 mm at elevated and peak regions [*Konnert*, 2004; *Kraller et al*., 2012]. Despite the dense station network in the catchment, the latter figure is still subject to high uncertainties because of the usual measurement errors [*Sevruk*, 1985] and a limited number of meteorological stations at high elevations. The *Berchtesgaden National Park*, which covers an area of 210 km^2^, is situated within the borders of the catchment.

## 3. Hydrological Model: Data, Setup, and Model Runs

[7] The deterministic, distributed hydrological model Water Balance Simulation Model ETH (WaSiM-ETH) [*Schulla and Jasper*, 2000] is applied to simulate water flux and storage terms in the catchment. The model comprises process-based, mass-conserving algorithms for the components of the terrestrial water cycle. Details can be found in the technical model description [*Schulla and Jasper*, 2000]. The model was applied successfully to Alpine catchments in previous studies by *Gurtz et al*. [2003], *Verbunt et al*. [2003], *Jasper et al*. [2004], *Kunstmann and Stadler* [2005], *Kunstmann et al*. [2005], and others. The required data to run the model consist of spatially distributed data sets to describe topography, land use, and soil parameters as well as station measurements of meteorological variables.

### 3.1. Hydrometeorological Data

**Figure 1:**
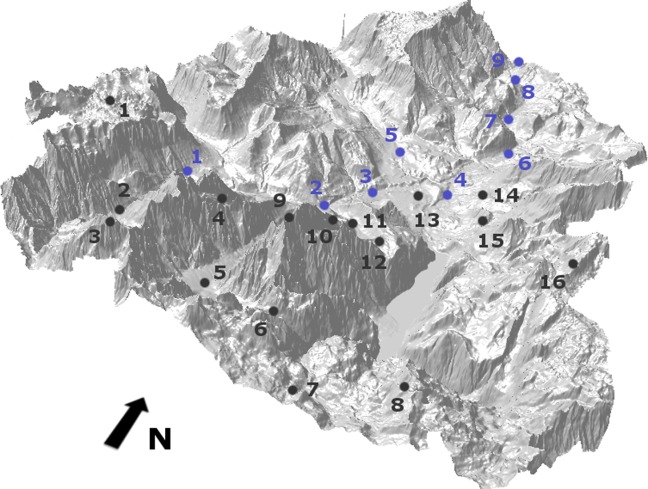
DEM (spatial resolution: 10 m) of the study area and positions of automatic meteorological stations (black) and runoff gauges (blue), listed in Tables 1 and 2.

[8] The meteorological data sets were recorded by 34 meteorological stations, of which 20 are operated automatically and provide hourly values of main meteorological variables. Fourteen of them are mechanical stations that provide daily measurements of precipitation. Six of the stations are situated in Austrian territory and operated by the *Central Institute for Meteorology and Geodynamics*. The stations in German territory are operated by the *Berchtesgaden National Park Administration*, the *Administration Union of the Berchtesgaden-Koenigssee Region*, the *Bavarian Avalanche Warning Service*, and the *German Weather Service* (Table 1). Figure 1 shows the locations of the 16 automatic stations within the modeling domain. All data were sampled every 10 s and recorded every 10 min. Recordings are then aggregated to hourly values (i.e., average for temperature, humidity, wind speed, radiation, and atmospheric pressure; total for precipitation). The daily precipitation data of the 14 mechanical stations are disaggregated to hourly values using the hourly measurements of the automatic stations. The study reported here is based on station data from 2001 to 2010.

**Table 1:** Altitude, Set of Recorded Parameters, and Temporal Resolution for the Meteorological Stations of the Automatic Network in the Berchtesgaden National Park^a^

ID	Station	Altitude (MSL)	Parameters	Temporal. Resolution	Operator
1	Reiteralm 1	1753 m	T, H, WS, WD	10 min	LWD
1	Reiteralm 2	1679 m	T, H, TS, SD	10 min	LWD
1	Reiteralm 3	1611 m	T, H, P, GR, RR, SD	10 min	LWD
2	Hinterseeau	839 m	T, H, WS, WD, GR, RR, SD	10 min	NPV
3	Hinterberghorn	2270 m	T, H, WS, WD, GR, RR	10 min	NPV
4	Blaueis	1651 m	T, H, WS, WD, GR, RR, SD	10 min	NPV
5	Brunftbergtiefe	1238 m	T, H, P, WS, WD, GR, RR, SD	10 min	NPV
6	Trischübel	1764 m	T, H, P, WS, WD, GR, RR, SD	10 min	NPV
7	Steinernes Meer	1900 m	T, H, P, WS, WD, GR, RR, SD	10 min	NPV
8	Funtenseetauern	2522 m	T, H, WS, WD	10 min	LWD
9	Watzmanngrat	2630 m	T, H, WS, WD, GR, RR	10 min	LWD
10	Watzmannhaus	1919 m	T, H, WS, WD, GR, RR	10 min	LWD
11	Falzalm	1484 m	T, H, P, WS, WD	10 min	LWD
12	Kühroint	1407 m	T, H, P, WS, WD, GR, RR, TS, SD, SWE	10 min	LWD
13	Schönau	617 m	T, H, P, GR, DR, SS, WS, WD, AP	10 min	DWD
14	Höllgraben	640 m	T, H, P	10 min	LWD
15	Jenner	1219 m	T, H, P, WS, TS, SD	10 min	LWD
16	Schlunghorn	2155 m	T, H, WS, WD, GR, RR	10 min	NPV
	Lofer	625 m	T, P, H, WS, WD, GR, SS, AP	1 h	ZAMG
	Loferer Alm	1623 m	T, P, H, WS, WD, GR, SS, AP	1 h	ZAMG
	SBG Flughafen	430 m	T, P, H, WS, WD, GR, SS, AP	1 h	ZAMG
	Schmittenhöhe	1973 m	T, P, H, WS, WD, GR, SS, AP	1 h	ZAMG
	Mülldeponie Winkel	699 m	P	1 day	NPV
	Königsberg Pegel	1532 m	P	1 day	NPV
	Schapbach	953 m	P	1 day	NPV
	Kühroint (mech.)	1418 m	P	1 day	NPV
	Lahneralm	1240 m	P	1 day	NPV
	St. Bartholomä	604 m	P	1 day	NPV
	Wimbachschloss	926 m	P	1 day	NPV
	Brunftbergtiefe (mech.)	1238 m	P	1 day	NPV
	Auf dem Gries	1435 m	P	1 day	NPV
	Bindalm	1119 m	P	1 day	NPV
	Eckau	1015 m	P	1 day	NPV
	Lahnwaldfütterung	840 m	P	1 day	NPV
	Mittereis	1325 m	P	1 day	NPV
	Halsalm	1088 m	P	1 day	NPV

a Accuracy of the recordings used in the modeling is 0.3 m s^−1^ (wind speed), 0.3°C (temperature), 1% (humidity), 5% (global radiation), and <0.4% (precipitation), respectively. Accuracy of the snow pillow recording SWE is 0.25% and of the ultrasonic ranger measuring snow depth is 0.1%. All accuracies are according to the technical specifications of the manufacturers. T, air temperature; H, relative humidity; WS, wind speed; WD, wind direction; SD, snow depth; SWE, snow water equivalent; SS, sunshine duration; GR, global radiation; DR, direct radiation; RR, reflected radiation; P, precipitation; AP, atmospheric pressure at sea level; TS, surface temperature; LWD, Bavarian Avalanche Warning Service; NPV, Administration Berchtesgaden National Park; ZAMG, Central Institute for Meteorology and Geodynamics; DWD, German Weather Service.

[10] The point measurements of meteorological variables are spatially distributed to each grid cell of the model domain. Air temperature, humidity, wind speed, and precipitation are interpolated with an elevation-dependent regression function considering individual regression parameters for each time step and three different elevation layers. This allows for the detection and reproduction of atmospheric inversion conditions. Measured global radiation is used to calculate cloudiness by comparison to modeled-topography- and time-dependent potential global radiation. Snow water equivalent (SWE) at the station *Kühroint* is measured by means of a snow pillow. Snow depth is captured with an ultrasonic ranger.

[11] Measurements of runoff are provided by the *Water Authority Traunstein* for eight gauges in the region (Table 2). The discharge time series of the Austrian gauge *St. Leonhard* is provided by the *Hydrographic Service Salzburg*. Table 2 shows the name of the gauges, the associated streams, and the areas of the respective watersheds. Figure 1 displays the locations of the gauges. Runoff data are available from 2001 to 2007.

**Table 2:** Gauging Stations, Stream Channels, and Watershed Areas of the Subbasins

ID	Gauge/subbasin	Stream	km^2^	%
1	Hintersee	Ramsauer Ache	42.69	9.9
2	Ramsau	Wimbach	35.72	8.3
3	Ilsank	Ramsauer Ache	46.90	10.8
4	Schwöb	Königsseer Ache	166.47	38.5
5	Stanggass	Bischofswieser Ache	47.45	11.0
6	Kläranlage	Berchtesgadener Ache	33.58	7.8
7	Almbachmühle	Almbach	9.83	2.3
8	Schaden	Rothmannbach	8.00	1.8
9	St. Leonhard	Berchtesgadener Ache	41.68	9.6
			432.32	100

### 3.2. Spatially Distributed Data Sets

[12] The hydrological model applied requires a set of spatially distributed input data. A digital elevation model (DEM) in a spatial resolution of 50 m is used to drive the model and derive all required topographic parameters. A regional soil map is provided by the *Bavarian Environment Agency*. During preprocessing, the soil types of the soil map are aggregated to 12 classes based on the classification of the *German Soil Map*. Land use is classified based on the project Alpine Habitat Diversity (HABITALP) [*Lotz*, 2006] by interpretation of color-infrared aerial images. Habitats of all protected areas in the Alps are mapped on the basis of a jointly defined interpretation key. For the parts of the catchment situated in Austria, the HABITALP classification is not available. The land use map is complemented by data from the Corine Land Cover project (http://www.eea.europa.eu/data-and-maps/) of the European Environment Agency.

### 3.3. Setup and Model Runs

[13] WaSiM-ETH model runs are performed for the period from 2001 to 2010 with a horizontal resolution of 50 m and a temporal resolution of 1 h model time step. Evapotranspiration is determined by the Penman-Monteith model [*Penman*, 1948]. Water flow in the unsaturated zone is calculated by solving the 1-D *Richards* [1931] equation. The saturated zone is represented by a 2-D groundwater flow model. Additional information on model setup and performance is listed in *Kraller et al*. [2012]. To investigate the influence of the different snow process parameterizations on discharge modeling, four model runs with different combinations of snow cover modeling methods (Table 6) are performed. The different approaches are explained in detail in the following section.

## 4. Snow Cover Evolution Modeling

[14] The main focus of this work is modeling snow cover distribution by methods of different complexity. Besides the existing snow module of WaSiM-ETH, which is based on a traditional degree-day approach, methods were further developed and integrated that have proven to be robust representations of Alpine snow processes. Model results of mountain snow cover evolution simulated by the formulations used in this study were previously compared to measurements in a variety of geographical regions, including analyses on a regional scale using snow cover data derived from satellite data [*Strasser and Mauser*, 2001; *Strasser et al*., 2002, 2004; *Prasch et al*., 2007; *Strasser*, 2008; *Strasser et al*., 2008; *Strasser and Marke*, 2010; *Strasser et al*., 2011; *Zappa et al*., 2003]. Model results were additionally validated within the framework of the two international snow model intercomparison projects SnowMIP [*Etchevers et al*., 2004] and SnowMIP2 [*Rutter et al*., 2009].

[15] The following formulations and modules were implemented in WaSiM-ETH. (1) To determine melt as well as sublimation and resublimation rates, an energy balance scheme [*Anderson*, 1973; *Strasser et al*., 2008; *Weber*, 2008; *Strasser and Marke*, 2010] was applied. (2) To account for lateral, gravitational snow transport processes, the algorithm according to *Gruber* [2007] was implemented. (3) To assess wind-driven snow redistribution, a method based on the idea of *Winstral and Marks* [2002] was further developed. All approaches were fully integrated in the snow module of WaSiM-ETH.

### 4.1. Snow Accumulation

[16] Snow accumulation is controlled by snow precipitation, resublimation, and lateral snow transport. Snow precipitation is estimated for each grid cell using the interpolated air temperature during the event and a transition range with liquid and solid precipitation. The fraction of snow is given by
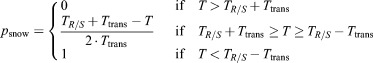
(1)with the fraction of snow in total precipitation 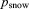 air temperature *T*, temperature for 50% snowfall fraction 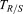, and half of the temperature transition range 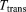 [*Schulla and Jasper*, 2000]. In this study, 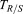 is set to 273.66 K and 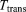 to 1 K. Resublimation is explicitly determined during the energy balance calculation. It is neglected when using a temperature index approach, as this method determines mass losses only. Lateral transport mechanisms change snow accumulation rates according to the formulations in section 4.3.

### 4.2. Snow Ablation

[17] Snowmelt, sublimation, and lateral transport are the processes that lead to the ablation of the snow pack at a certain location. Snowmelt and sublimation are explicitly described by the implemented energy balance approach. When using a temperature index approach, snow sublimation can be regarded as inherently included in the calibrated melt or ablation factor. Generally, in the region investigated sublimation losses are small compared to snowmelt [*Strasser*, 2008]. Furthermore, ablation processes are controlled by the lateral transport mechanisms described in section 4.3.

#### 4.2.1. Temperature Index Approach

[18] Temperature index approaches, e.g., the degree-day method, are widely used for modeling snowmelt. In WaSiM-ETH it is implemented by
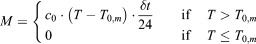
(2)where *M* is the melting rate per time step, 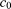 is a temperature-dependent ablation factor, *T* is the air temperature, 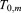 is the lower temperature limit for snowmelt, and 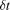 is the model time step in hours [*Schulla and Jasper*, 2000].

[19] In this study, 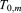 is set to 273.16. The degree-day factor 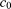 was calibrated by *Marx et al*. [2008] in an adjacent catchment with similar characteristics and is kept constant at 2.5 mm d^−1^ K^−1^, this value being in a valid range for Alpine catchments [*Hock*, 2003]. Equation (2) disaggregates the daily melt rates into hourly values by using hourly air temperatures and a scaled degree-day factor.

#### 4.2.2. Energy Balance Approach

[20] The energy balance of the snow surface is calculated hourly considering short-wave and long-wave radiation, sensible and latent heat fluxes, energy conducted by solid and liquid precipitation, (re)sublimation, and a constant energy input originating from the soil heat flux. The snow pack is regarded as a single layer of homogeneous snow beneath the surface for which the energy balance is calculated. A distinction is made using air temperature as a proxy between melting conditions (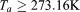) and nonmelting conditions (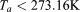), with 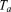 being the current air temperature. In the first case, a snow surface temperature of 273.16 K is assumed and melting occurs, if the computed energy balance is positive. In the case of nonmelting conditions, an iterative scheme to close the energy balance is applied, where the snow surface temperature is adjusted and the long-wave emission and turbulent fluxes are recalculated until the energy balance residual equals zero [*Strasser*, 2008; *Weber*, 2008; *Strasser et al*., 2011].

[21] The decision between melting and nonmelting conditions by using air temperature as a proxy has proven to be a stable approach when determining the energy balance of the surface [*Weber*, 2008]. For this application—runoff simulations on the regional scale—the processes in the underlying snow layers are only of interest in the respect that they can influence the energy balance of the surface by a potential cold content. The proxy approach has proven to be robust, as air temperature above a snow surface is controlled by the energy balance of the snow cover beneath [*Weber*, 2008]. However, situations might occur where air temperature exceeds 273.16 K, while the snow surface underneath is still below that threshold. In these instances, the assumption fails and the model possibly produces snow melt if the energy balance is positive.

[22] For a snow surface, the energy balance can be expressed as
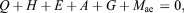
(3)where *Q* is the short-wave and long-wave radiation balance, *H* is the sensible heat flux, *E* is the latent heat flux, *A* is the advective energy supplied by solid or liquid precipitation, *B* is the soil heat flux, and 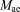 is the energy potentially available for melting during a given time step [*Anderson*, 1973]. All energy flux densities are expressed in W m^−2^. The soil heat flux *G* is assumed to be constant in space and time, as it is very small compared to other fluxes. It is set to 2 W m^−2^, this value being a robust average for mid-European Alpine sites [*Durot*, 1999]. The available energy for melting 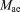 can be computed for the case of melting conditions (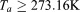). For this case, all fluxes are calculated with an assumed snow surface temperature of 273.16 K, and 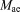 is the remainder of the energy balance equation. If 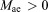, the amount of melting *M* in millimeters is calculated as
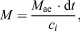
(4)where 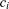 is the latent heat of fusion. The equations for calculations of sensible and latent heat fluxes can be found in *Kuchment and Gelfan* [1996], *Strasser* [2008], and *Strasser et al*. [2008]. The advective heat flux by precipitation is calculated using precipitation amounts and phase, as well as air temperature according to *Strasser* [2008]. For consistency, the snow cover energy balance model uses the same radiation model as the evapotranspiration module of the hydrological model documented by *Schulla and Jasper* [2000].

### 4.3. Lateral Redistribution of Snow

[23] Lateral redistribution of snow is a complex process on different spatial and temporal scales depending on a variety of controlling mechanisms. Two main drivers for lateral transport are topography and atmospheric boundary conditions, mainly actual surface wind speed and direction. This work aims at introducing and investigating modules to simulate snow redistribution not on an event basis, but on the catchment scale and in a model complexity that facilitates to reproduce the hydrologically relevant redistribution of water. This is implemented by the application of an algorithm to calculate gravitationally driven transport of snow in steep mountain topography and a parameterization of wind-driven snow redistribution.

#### 4.3.1. Gravitational Snow Transport

[24] Sliding of snow from steep walls and faces to the base of the slopes was identified to be an important process in modeling accumulation and ablation of snow in high mountain regions with steep terrain [e.g., *Dadic et al*., 2008; *Strasser*, 2008; *Bernhardt and Schulz*, 2010]. For distributed snow process modeling on the catchment scale and long-term simulations of high mountain snow cover dynamics and glacier response to climate change in Alpine regions, accounting for gravitational, lateral redistribution of snow is an indispensable prerequisite for obtaining realistic results for the simulated SWE development. An approach according to *Gruber* [2007] was implemented in the hydrological model to consider the gravitational transport of snow. It was tested in the studies by *Dadic et al*. [2008] and *Strasser* [2008]. *Bernhardt and Schulz* [2010] successfully implemented a similar approach in a distributed snow model. Our study presents the first implementation of such a method within a fully distributed, physically based water balance model.

[25] The approach is based on an analysis of the topography and the available mass input. It is a mass-conserving, multiple-direction flow propagation procedure that routes entrained snow masses along predefined flow couloirs derived from the DEM. In each cell, the fraction of mass 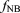 drained to a neighbor NB is a function of topography only and totals one over all neighboring cells in order to conserve mass. Deposition 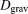 is limited by the local maximum deposition 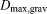 and determined by the available incoming mass 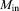 that is the sum of the received inflows from the neighboring cells. Potential surplus of sliding snow is transported into the neighboring cells. All masses are denoted as mm SWE. The flow 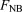 into each neighbor NB of a cell is given by the total outflowing mass of a cell 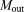 and the draining fractions 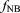:
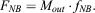
(5)

[26] The calculation of the draining fractions is explained in detail by *Gruber* [2007]. The total outflow of a cell is
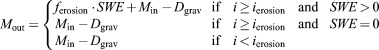
(6)with 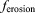 being an erosion factor depending on model time step, SWE being the snow storage in the current pixel, *i* being the local inclination, 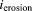 being a lower inclination limit for snow erosion, 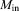 being the received inflow from all neighbors, and 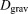 being the deposition in the pixel in millimeters.

[27] Deposition 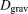 is controlled by the available mass and the local maximum deposition. The amount of snow deposited in a cell can be quantified as
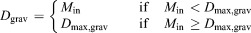
(7)with 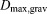 being the maximum snow mass that can be deposited within a single DEM cell during a slide event. The parameter 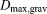 is determined by local inclination. The following simple linear function is used to quantify 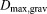 [*Gruber*, 2007]:
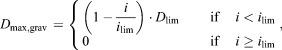
(8)where 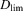 is an upper deposition limit, e.g., the maximum amount of snow that would be deposited on horizontal terrain, and 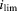 is an upper limiting angle above which all incoming sliding snow is transported downslope. 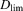 is related to the model time step.

[28] The parameterization of the model depends on the model time step, the spatial resolution of the DEM, and local topography characteristics. Some values have to be derived empirically, and parameters have to be adapted to produce flow paths which are coherent with visual experience. For the current study, the model was initialized with the following settings: 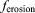 is 1% of the actual snow storage in a cell. If local inclination *i* exceeds the lower limiting angle 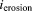 of 45°, snow is eroded (equation (6)). As a reasonable value for the threshold, above which incoming sliding snow is not deposited, but entrained further downslope (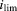, equation (8)), 55° was identified. Cells that are steeper than 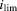 still receive snow by precipitation. They are constantly freed of snow by gravitational transport within a certain time period after a snowfall depending on 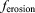.

[29] In this parameterization, both flow and deposition physics are not described explicitly but characterized by simple parameters. Since the effects of kinetic energy are not included, potential uphill flow of fast moving snow masses is neglected. Many other factors like stability criteria in the layers of the snow pack, type of underground, local-scale meteorological conditions, or external forces also are not considered here. Therefore, the timing of avalanches on an event basis cannot be predicted by this method, but the procedure allows for a hydrologically plausible redistribution of snow [*Strasser*, 2008].

#### 4.3.2. Wind-Driven Snow Redistribution

[30] A main driver of spatial heterogeneity of the snow cover in complex terrain is redistribution of snow caused by wind. Wind-driven snow redistribution is important to local and regional hydrology as well as to the assessment of the danger potential due to natural hazards, as wind-loaded slopes are often avalanche-prone locations due to big snow masses paired with instabilities within the snow pack. Apart from the wind impact on the energy balance of the snow pack, three major interaction mechanisms of wind and snow shape the complex snow cover distribution in mountainous terrain: preferential deposition of snow precipitation, wind-driven redistribution of previously fallen snow, and the effective sublimation of suspended snow into the atmosphere [*Lehning et al*., 2008; *Mott et al*., 2008; *Dadic et al*., 2010a]. These processes lead to a highly variable distribution of snow on different spatial scales [*Blöschl and Kirnbauer*, 1992; *Pomeroy et al*., 1998; *Liston and Sturm*, 1998; *Lehning et al*., 2008; *Strasser*, 2008; *Bernhardt et al*., 2010; *Dadic et al*., 2010a,2010b; *Mott et al*., 2010].

[31] Several methods of different complexity have been developed to account for wind-driven snow transport and redistribution by *Liston and Sturm* [1998], *Winstral and Marks* [2002], *Lehning et al*. [2008], *Mott et al*. [2008], *Raderschall et al*. [2008], *Bernhardt et al*. [2009, 2010], and *Dadic et al*. [2010a]. In this study, a simple parameterization is used to capture all the wind-driven snow processes described and to estimate and reproduce the result of their interaction. The basic principle is the extraction of locations that are sheltered from or exposed to wind by a topography analysis and a respective correction of snow precipitation. This approach is in line with the idea of *Winstral and Marks* [2002]. The topography analysis is based on a modified algorithm by *Corripio* [2003] that calculates the sky view factor (SVF). The routine was modified to determine a partial, directed SVF (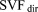). The correction is done by specification of sectors representing prevailing main wind directions. Snow precipitation is corrected by
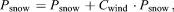
(9)where 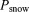 is the solid precipitation (snow). The correction factor 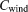 is calculated for each cell of the domain:
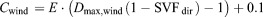
(10)with a (linear) elevation weighting factor *E*, the directed SVF 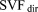, and the maximum possible deposition 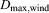. The parameter *E* ranges from 0 at the lowest elevated pixel to 1 at the highest pixel and linearly scales the amount of snow redistribution. This scaling is based on the assumption that the lower wind speeds at lower elevations reduce redistribution. In this implementation, the calculation of 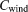 results in values between −0.82 and +1.84. This method certainly has drawbacks and limitations, as it is a simple estimation that should not be expected to produce highly accurate values for redistributed snow masses. Accuracy in modeling these processes on the regional scale additionally is highly depending on spatial and temporal resolutions. Benefits of the method are its computational efficiency and the transferability without the need for complex wind field input data. In contrast to *Winstral and Marks* [2002], the application is restricted to lateral snow redistribution and does not adjust wind speed values for the computation of turbulent fluxes.

## 5. Results and Discussion

### 5.1. Snow Model Performance

#### 5.1.1. Performance on the Point Scale

**Figure 2:**
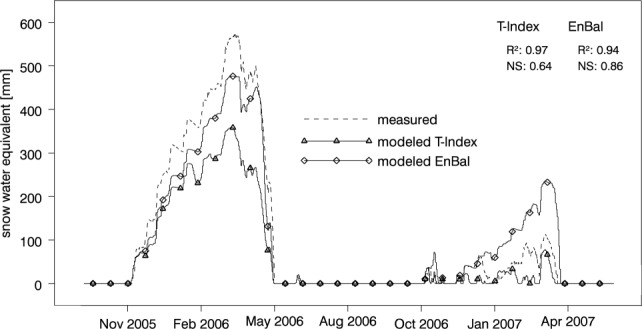
Seasonal snow cover evolution at the Kühroint station site (1407 m MSL) as modeled and recorded with a snow pillow during the winter seasons 2005/2006 and 2006/2007.

**Figure 3:**
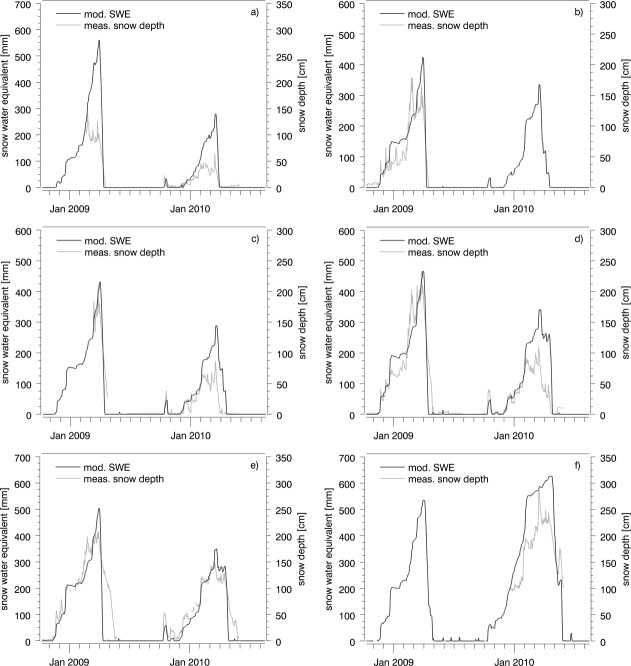
Comparison of seasonal modeled SWE evolution (energy balance approach without lateral redistribution) and measured snow depth values at six stations for the winter seasons 2008/2009 and 2009/2010. Stations are from top left to bottom right: (a) Hinterseeau 839 m MSL, (b) Jenner 1219 m MSL, (c) Brunftbergtiefe 1238 m MSL, (d) Kühroint 1407 m MSL, (e) Blaueis 1651 m MSL, and (f) Steinernes Meer 1900 m MSL. For stations (b) and (f), no data were available for 2008/2009 and 2009/2010, respectively.

[32] Figure 2 shows the seasonal snow cover evolution at the station Kühroint during two winters. Simulated and observed values of SWE are compared. Both the temperature index and energy balance approach perform differently during the two winter seasons. The course of SWE during the snow-rich winter 2005/2006 is better reproduced by the energy balance approach and underestimated by the temperature index method. For the winter season 2006/2007, which is characterized by an exceptionally small amount of snow, the temperature index method performs slightly better. The energy balance approach overestimates SWE in this season. This is mainly caused by missing the melt out event in December 2006. However, these validation results are based on the measurements and simulations at a single point in the catchment, which means at one out of 172,959 model grid cells. Moreover, the location of the station is not representative for the whole study area. It can be assumed that these results would strongly vary within the complex terrain of the catchment, the key characteristics for snow cover development being very inhomogeneous, e.g., exposure, altitude, steepness, surrounding terrain, or land cover. The performance of the temperature index model in Figure 2 could have been improved at the point scale by additional calibration by different factors for single winter seasons. In addition to the SWE data at the station Kühroint, measurements of snow depth are available at several locations in the catchment. As the density of the snow cover and snow depth values are not computed by our model system, we compare SWE with measured snow depths to allow an evaluation of the dynamic behavior. Figure 3 presents a comparison of measured snow depth and modeled SWE at six stations that cover an elevation range from 839 to 1900 m MSL for the winter seasons 2008/2009 and 2009/2010 to further validate the implemented energy balance approach and the general model setup.

[33] The observed timing of first seasonal snowfall and melt out dates are well produced throughout this large elevation range by the model. At the station *Blaueis* (Figure 3e), the snow cover melts off faster than observed resulting in an underestimated snow cover duration. This deviation might also be a caused by the specific location of the station possibly measuring at a point with unrepresentative large snow amounts, as snow cover duration significantly differs from the other stations. The timing of single snowfall events is mostly modeled correctly. This can be seen from the corresponding time periods of increasing SWE and snow depth values. The short period of preseasonal snow coverage in October 2009 is reproduced at all stations where observations are available (Figures 3a, 3c, 3d, and 3e). The model seems to overestimate the amount of snow at the lowest elevated station *Hinterseeau* (Figure 3a). This can explained by an underestimation of snowfall amounts and possibly by higher snow densities at the station characterized by warmer climatic conditions. Regarding the total catchment, snow cover duration and amounts are modeled with a satisfying performance, even though there are model errors at some stations. It is noted that there are uncertainties in this comparison induced by scale difference. The observations are valid for one specific point, whereas the modeled values represent a mean value of a cell with a surface area of 2500 m^2^. Moreover, using the presented methods for spatial interpolation of meteorological forcing variables (section 3.1), the measured driving data are not necessarily reproduced at a station during model runtime. Additionally, there are uncertainties when determining the precipitation phase (equation (1)). As the used energy balance model is a relatively simple one layer approach using a proxy assumption (section 4.2.2), future research may show if model performance can be improved by using a more complex energy balance approach or by using an even more complex multilayer snow model.

#### 5.1.2. Performance on the Catchment Scale

[34] The modified snow module calculates the energy balance of the snow cover considering topography-dependent radiation fluxes as well as lateral snow transport processes. The scheme explicitly describes short- and long-wave radiation fluxes, turbulent latent and sensible heat exchange at the snow cover, advective energy by precipitation, as well as a constant soil heat flux. It also accounts for mass changes accompanying the latent heat flux. This leads to a topography-driven distribution of the snow ablation processes depending on exposure, slope, and shading effects.

[35] The consideration of lateral snow transport processes covers two mechanisms: snow redistribution by snow slides on very steep slopes and wind-induced snow transport. A simple mass-conserving approach was applied for the simulation of snow slides and a parameterization for the estimation of wind-induced snow transport based on an analysis of the DEM that extracts exposed and sheltered locations.

**Figure 4:**
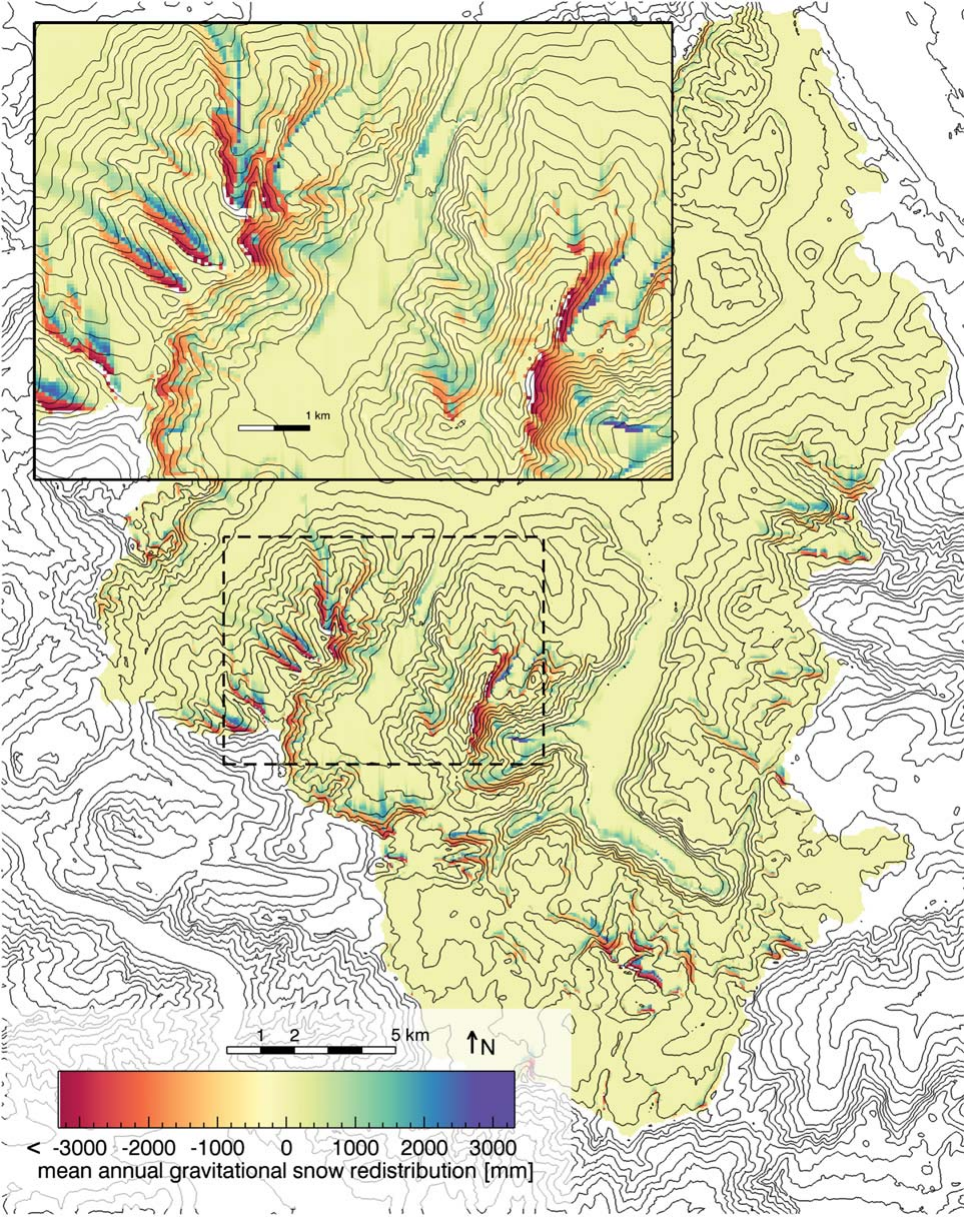
Simulated mean annual lateral snow redistribution by gravitational snow transport when additionally accounting for wind-driven redistribution (see details in the text). Red regions show erosion, and blue regions show accumulation zones. Minimum values range down to −4448 mm at very steep slopes with high snow precipitation. Contour lines denote elevation levels of 150 m (100 m in the enlarged view). The dashed rectangle demarcates the area shown in the enlarged view.

[36] As a result of introducing gravitational slides, snow is removed from steep rock faces and accumulated at the base of the slopes. Erosion or ablation rates at the steepest slopes with highest snow precipitation reach up to 2023 mm SWE per winter. These areas are high elevated peaks with steep rock faces. The deposition rates are 2957 mm additional SWE per year at some locations. Accordingly, ranges of gravitational snow redistribution were simulated by *Strasser* [2008] and *Bernhardt and Schulz* [2010]. When the snow slide module is combined with the parameterization of wind-driven snow distribution, these maximal values increase to 4448 mm erosion and 3311 mm deposition (Figure 4), because of higher incoming snow masses at the respective elevated steep slopes on leeward ridge sides.

**Figure 5:**
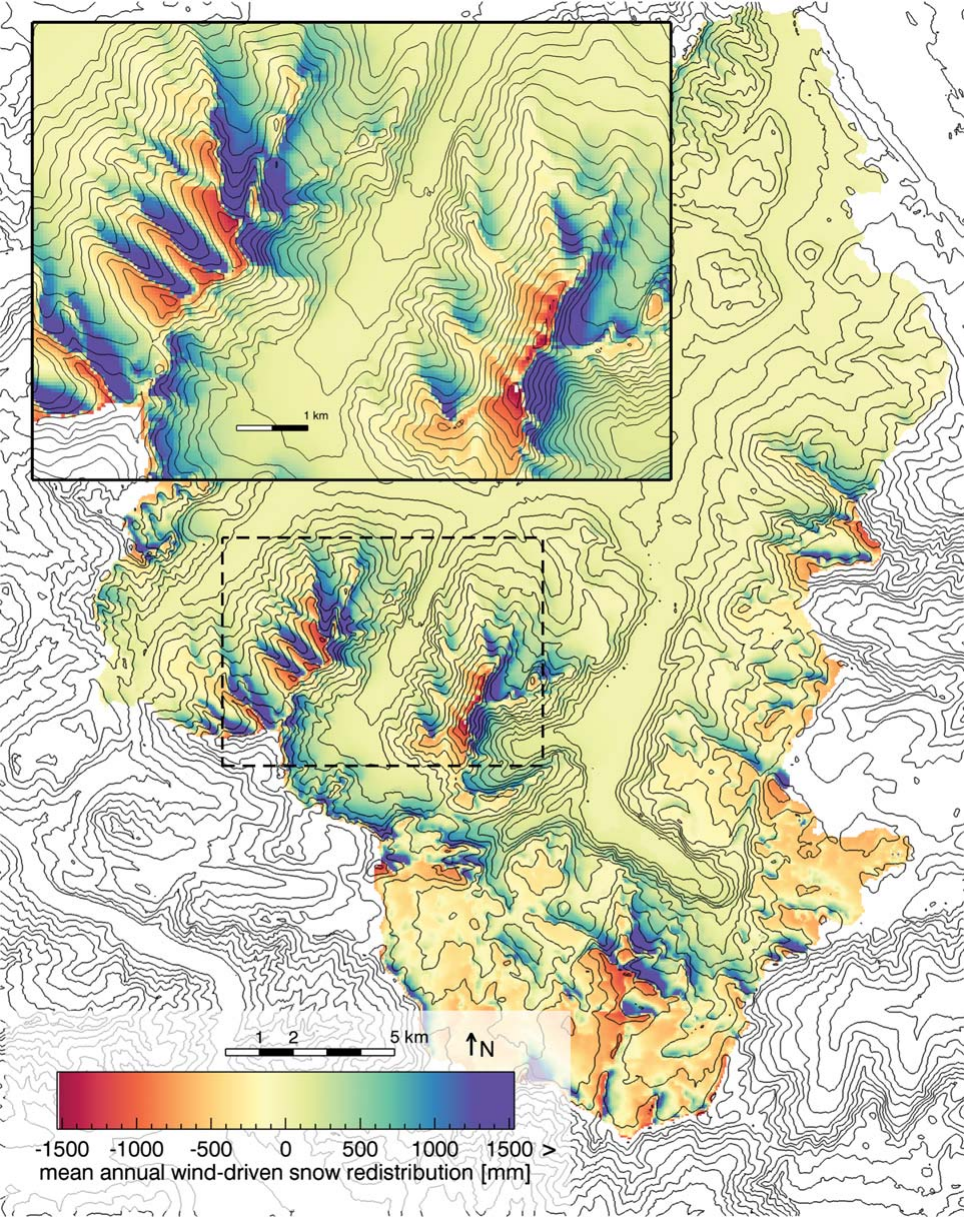
Simulated mean annual lateral wind-driven snow redistribution. Main wind direction is southwest. Red regions show erosion, and blue regions show accumulation zones. Maximum values range up to 3214 mm but are limited to small, high elevated, leeward locations. Contour lines denote elevation levels of 150 m (100 m in the enlarged view). The dashed rectangle demarcates the area shown in the enlarged view.

[37]Figure 5 illustrates the amount of snow that is redistributed by the parameterization of wind-driven snow transport. In this study, the directed SVF is kept constant and was calculated for a sector of 90° from south to west, which represents the main wind directions during winter season. The direction of the sector is based on a station analysis (not presented here) that matches an assessment by *Konnert* [2004]. This constant directed SVF limits the redistribution structure to a constant field. Depending on data availability regarding main wind directions, the approach could be extended by implementing time-varying parameter fields. As described earlier, the redistribution is implemented as a correction of precipitation before the modeling process. It is important to know that this approach is not mass conserving in the sense of eroded snow mass having to be equivalent to the deposited snow mass in total. Conservation of mass is not a prerequisite here, as the approach is to correct snow precipitation before the modeling process. This can be regarded as a sophisticated method of spatial interpolation of snow precipitation measured at the stations to the model grid. Such spatial interpolation methods in general are not mass-conserving. In this implementation, a pixel receives on average 27.8 mm additional snow precipitation per year (+5.2% of total snow precipitation).

[38] The mean annual correction of snow precipitation in the domain with an assumed main wind direction sector of southwest is presented here. Station data show that this is the prevailing wind direction during winter seasons in the region (not presented here). Slopes oriented to this sector, as well as high elevated ridges and peaks, are windward locations and receive up to 1574 mm less snow precipitation per winter season. In contrast to this, sheltered regions at leeward locations facing the northeast gain additional snow precipitation of up to 3214 mm. The patterns and spatial distribution are similar to the results obtained by *Bernhardt et al*. [2010] in their study, and the values of redistribution are in a similar order. They model maximum gains of SWE by wind-induced snow transport of 2140 mm in a case study at the *Blaueisgletscher*. The amounts computed by the presented parameterization are higher and range up to 2634 mm at this location. *Bernhardt et al*. [2012] compare seasonal snowmelt differences between a model run with and without the consideration of lateral snow transport. They present a spatial distribution of values ranging from maximal losses of 1600 mm SWE to maximal gains of 5000 mm by simulating lateral snow processes. In the same comparison, we assess similar values of maximal 1989 mm losses and maximal 5155 mm gains in seasonal snowmelt distributed in a matching spatial pattern.

[39] Calculated with the temperature index method, snow cover evolution depends on the precipitation amount and air temperature, and therefore, its duration increases with elevation. The energy balance approach in combination with lateral redistribution processes results in an exposition-dependent snow cover duration with a maximum at high elevated, northern-oriented, shaded, and sheltered areas at the foot of steep faces (Figure 8), where the accumulation is large due to high snowfall rates and incoming mass from snow slides and the energy input for ablation by solar radiation and sensible heat is limited.

**Figure 6:**
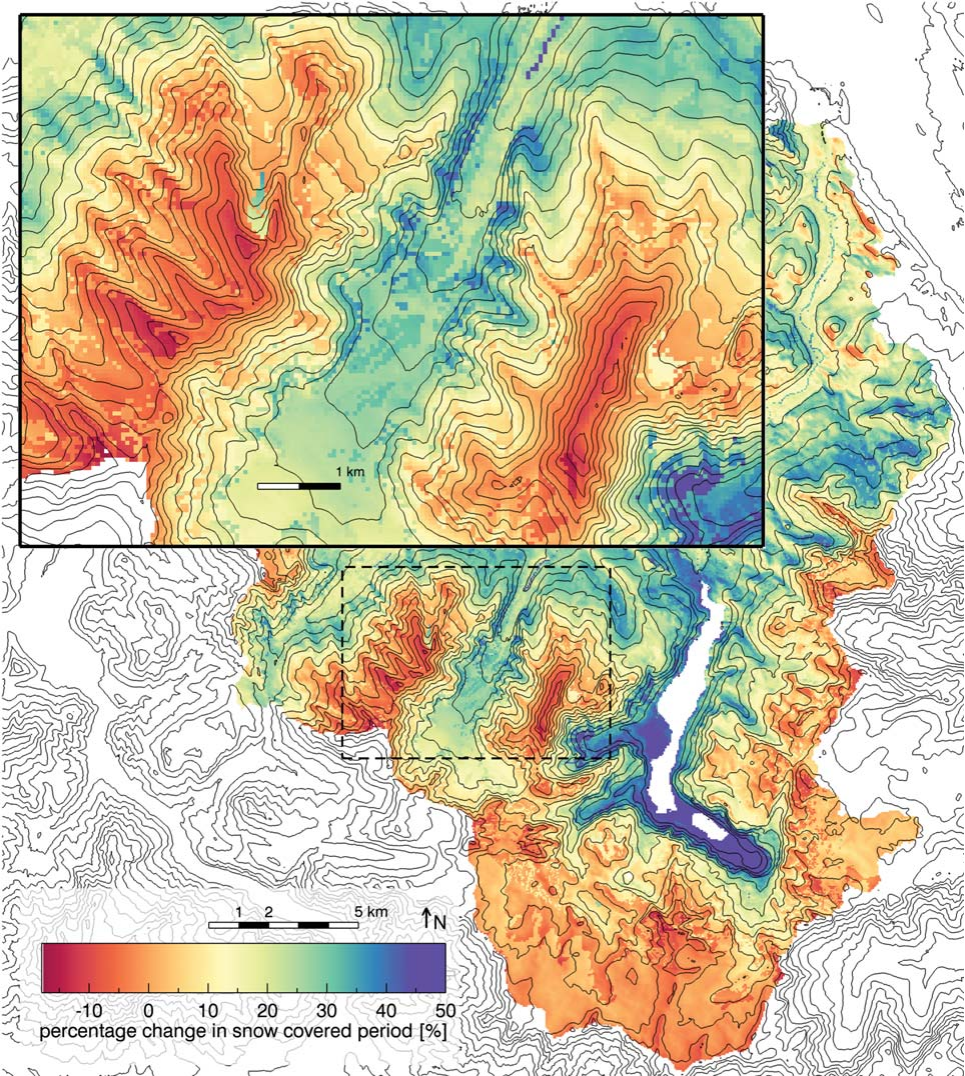
Difference in modeled mean annual snow cover duration between temperature index (TI) and energy balance (EB) approach (percentage change EB − TI). Contour lines denote elevation levels of 150 m (100 m in the enlarged view). The dashed rectangle demarcates the area shown in the enlarged view.

[40] Figure 6 illustrates the differences between the snow pack development simulated by means of a temperature index model and that modeled by the energy balance calculation, both without accounting for lateral snow transport. The range in gain and loss of snow cover duration is significant. The values range from 58 days less snow coverage per year to additional 45 snow-covered days at certain locations on average. The respective percentage change of snow cover duration at each cell is displayed in Figure 6 and ranges from −18% to +50%. The maximal losses in modeled snow cover duration can be found at high elevated, southern-oriented ridge sites because of the explicitly modeled incoming radiation energy. When using a temperature index approach, melting is underestimated particularly in high elevated, south-facing slopes with high radiation energy input and small average air temperatures.

**Figure 7:**
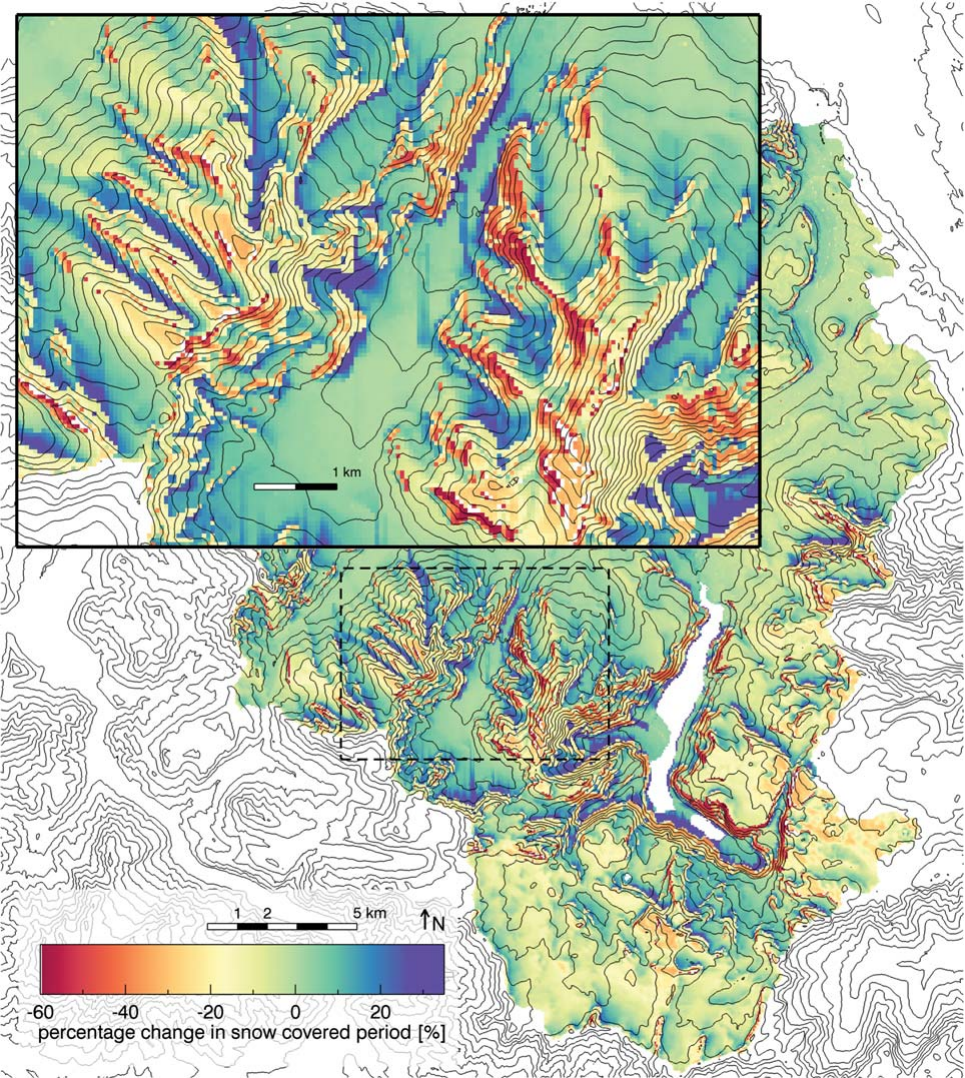
Difference in modeled mean annual snow cover duration due to lateral snow transport (percentage change “EB and lateral redistribution” − “EB without lateral redistribution”). Contour lines denote elevation levels of 150 m (100 m in the enlarged view). The dashed rectangle demarcates the area shown in the enlarged view.

[41] The effects of additionally accounting for lateral transport processes are shown in Figure 7, which presents the percentage change in snow cover duration by introducing the simulation of gravitational slides and wind-driven snow redistribution. Values range from −59% to +34% of change in mean snow cover duration. The maximal absolute changes are a reduction of 77 days per season and an increase of 57 days at certain cells. The maximal losses in modeled snow cover duration can be found at high elevated, exposed, steep ridge sites that are oriented to the main wind direction. These areas are blown free of snow by wind and lose snow masses by gravitational slides. The maximal gains in snow-covered days are located at leeward ridge sites and at the foot of steep faces, where snow is transported to by the snow slide algorithm.

**Figure 8:**
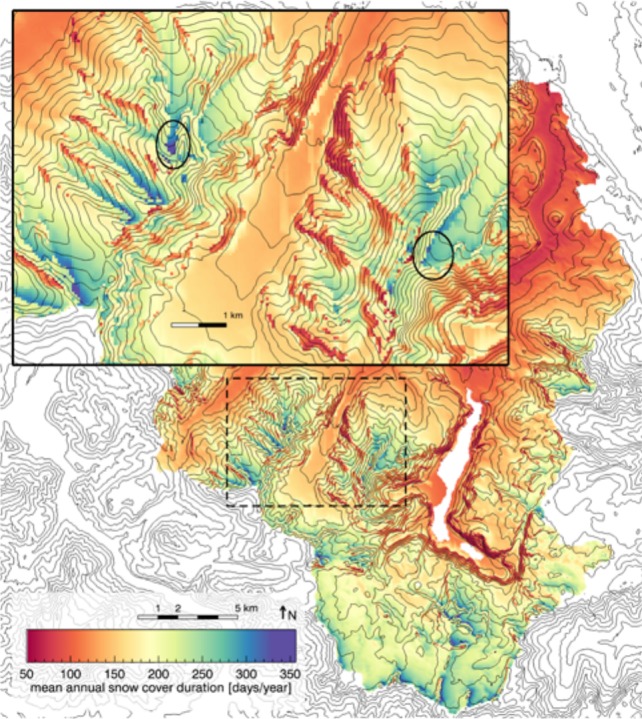
Modeled mean annual snow cover duration using the energy balance approach and simulating lateral redistribution of snow. Contour lines denote elevation levels of 150 m (100 m in the enlarged view). The dashed rectangle demarcates the area shown in the enlarged view. The locations of the two small glaciers in the catchment that are referred to in the text are indicated.

[42] In summary, maximum values of snow cover duration appear at the base of steep slopes, where the snow mass input is substantially increased by lateral transport mechanisms (Figure 7). Moreover, these locations are often shaded by surrounding steep faces. Low elevated, northern-oriented, shaded slopes generally show an increased snow cover duration when using the energy balance approach, independently of snow input by lateral transport (Figure 6). This is due to the consideration of the reduced radiative energy income in these locations. In contrast to this, the temperature index approach determines snow cover duration independently of exposure, if it is not implied in air temperature interpolation routines. The areas with a maximum snow cover duration of nearly 360 days per season (Figure 7) fit the locations where the perennial firn fields (*Schöllhorneis*, *Eiskapelle*), and glaciers are situated in the catchment (*Watzmanngletscher* and *Blaueisgletscher*). These realistic maximum values and particularly correct locations are not simulated by using the simple temperature index routine.

#### 5.1.3. Spatial Validation

**Figure 9:**
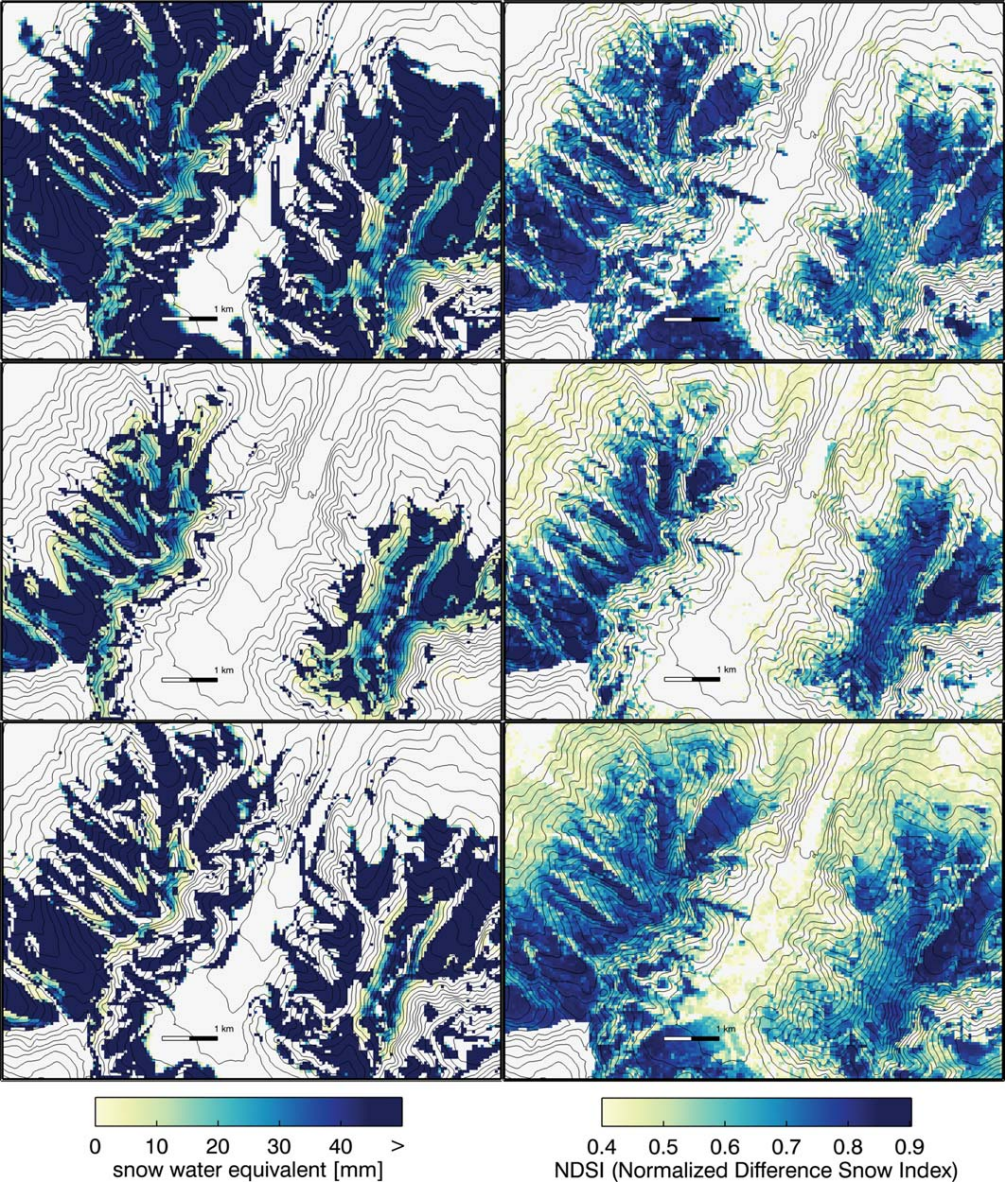
(left) Modeled SWE using the energy balance calculation including lateral snow transport compared to (right) NDSI from top to bottom on 7 April 2002, 30 May 2004, and 1 May 2005. NDSI was extracted from Landsat ETM+ scenes (see details in the text). SWE on the according dates is shown on the left with saturation of the color scale at 50 mm to reveal spatial details at locations with low SWE values. Snow-free pixels are presented in grey color. Percentage of cells that match between observation and model is from top to bottom 71.1%, 82.55%, and 72.5%.

[43] A comparison of modeled SWE and Landsat ETM+ imagery classification is shown in Figure 9. To validate the implemented snow model approaches, observed and modeled snow coverage are compared on three specific dates of data recording. The Normalized Difference Snow Index (NDSI) [*Dozier*, 1989; *Hall et al*., 1995] was extracted from the Landsat ETM+ data. The data are available in a horizontal resolution of 30 m and were interpolated to match the 50 m model grid. The threshold of 0.4 [*Dozier*, 1989] is used as a lower boundary to identify snow-covered pixels. Figure 9 shows the full NDSI range above the threshold. Cells having NDSI values below the threshold are indicated in grey color. The probability of the existence of snow in a pixel increases with higher NDSI values. As there are still uncertainties in classifying snow in the Landsat ETM+ data and particularly in the distinction between snow and rock in shaded locations, this probability map is shown in addition to the definite snow or no-snow decision. Matching Figures 7 and 9, the small-scale regions with high incoming snow masses by gravitational slides, as well as snow-free ridges, can be identified clearly in the observed and modeled images. A percentage of cells that are in agreement in the Landsat ETM+ NDSI classification and the modeled field of snow coverage was calculated (Table 3). The performance for the region shown in Figure 9 ranges from 71.1% to 82.5% and for the entire catchment from 82.5% to 89.5% at the different dates analyzed. The higher values for the entire catchment can be explained by a large area being free of snow in the lower elevations.

**Table 3:** Percentage of Cells That Are in Agreement in the Landsat ETM+ Classification and the Modeled Field of Snow Coverage (Energy Balance Calculation and Lateral Snow Transport) at Three Different Dates for the Summit Section Depicted in Figure 9 and the Entire Catchment

	7 April 2002	30 May 2004	1 May 2005
Summit section	71.1	82.5	72.5
Total catchment	84.0	89.5	82.5

[44] To further quantify the spatial analysis, additional performance indices are presented in the following. The model of indices was introduced by *Aronica et al*. [2002] and applied by *Hunter et al*. [2005] and *Bernhardt et al*. [2012]. The equations to compute the performance measures 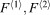, and 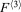 are
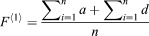
(11)
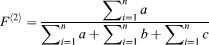
(12)
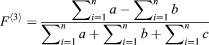
(13)with *a*, *b*, *c*, and *d* describing different combinations of modeled and observed binary patterns and *n* describing the number of pixels. The specific meanings of *a*, *b*, *c*, and *d* in this assessment are listed in Table 4.

**Table 4:** Contingency Table Listing the Parameters Used in Equations (11)–(13)

	Observed Snow	Observed No Snow
*Modeled* snow	*a*	*b*
*Modeled* no snow	*c*	*d*

[45] Table 5 presents the results for 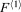
–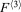 for the entire catchment and the three dates shown in Figure 9. All three indices are one if the modeled snow coverage perfectly matches the observed one. The parameter 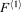 shows the same values as the percentage evaluation presented earlier (Table 3) as fractions of one. This performance index includes all pixels including the case *d* which represents pixels that are snow free in the model and in the observations. Since there are large areas that are low elevated and snow free in the NDSI classification as well as in the model simulation, this performance measure shows the highest values. 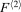 and 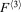 exclude these pixels and accordingly result in lower values. Generally, the performance is similar at the three dates representing spring snow coverage after three different winter seasons. Values for 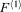 range from 0.83 to 0.90, for 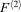 from 0.56 to 0.68, and for 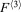 from 0.42 to 0.51. These values are in the same range as assessed by *Bernhardt et al*. [2012] in their study for one specific date.

**Table 5:** Performance Indices (Equations (11)–(13)) for the Three Evaluation Dates Comparing Landsat ETM+ NDSI Classification and Modeled Fields of Snow Coverage (Energy Balance Calculation and Lateral Snow Transport)

	7 April 2002	30 May 2004	1 May 2005
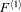	0.84	0.90	0.83
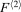	0.68	0.62	0.56
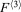	0.51	0.48	0.42

**Table 6:** Mean Nash-Sutcliffe Coefficients at the Gauge Hintersee, for the Mountainous Headwater Catchments (Subbasins/Gauges 1,2, and 4 in Table 2 and Figure 1) and for All Subbasins (Total) for the Period 2002–2007

Snow Module	Hintersee	Headwater Catchments	Total
T-Index	0.57	0.46	0.62
EnBal	0.62	0.49	0.63
EnBal + gravitational slides	0.65	0.51	0.64
EnBal + gravitational slides + wind redistribution	0.68	0.52	0.64

[46] The spatial distribution of snow coverage is refined by implementing the new snow model methods, resulting in a good agreement of modeled and observed snow distribution for simulations on the regional and catchment scale. However, a perfect match between modeled and real snow coverage or even SWE should not be expected, as some processes have not been addressed in the formulations, e.g., interaction between vegetation and snow or lateral heat exchange, and due to simplifications, restrictions in spatial resolution, and additional uncertainties, e.g., when measuring snow precipitation at the stations and spatially interpolating model input in the domain.

### 5.2. Influence on Runoff Generation and Streamflow Dynamics

**Figure 10:**
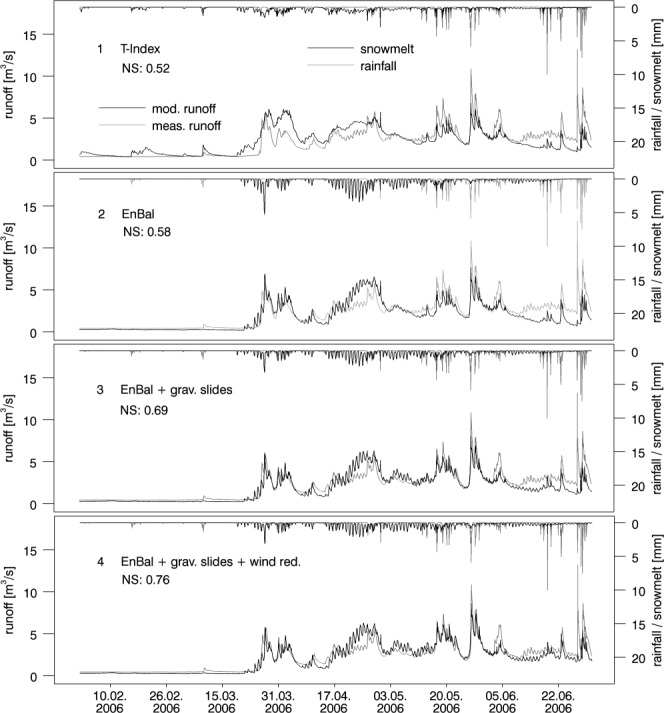
Modeled and measured runoff at gauge Hintersee from February to June 2006 and according to Nash-Sutcliffe coefficients (NS). Increasing snow model complexity from top to bottom: 1, temperature index; 2, energy balance; 3, energy balance and snow slides; 4, energy balance, snow slides, and wind redistribution.

**Figure 11:**
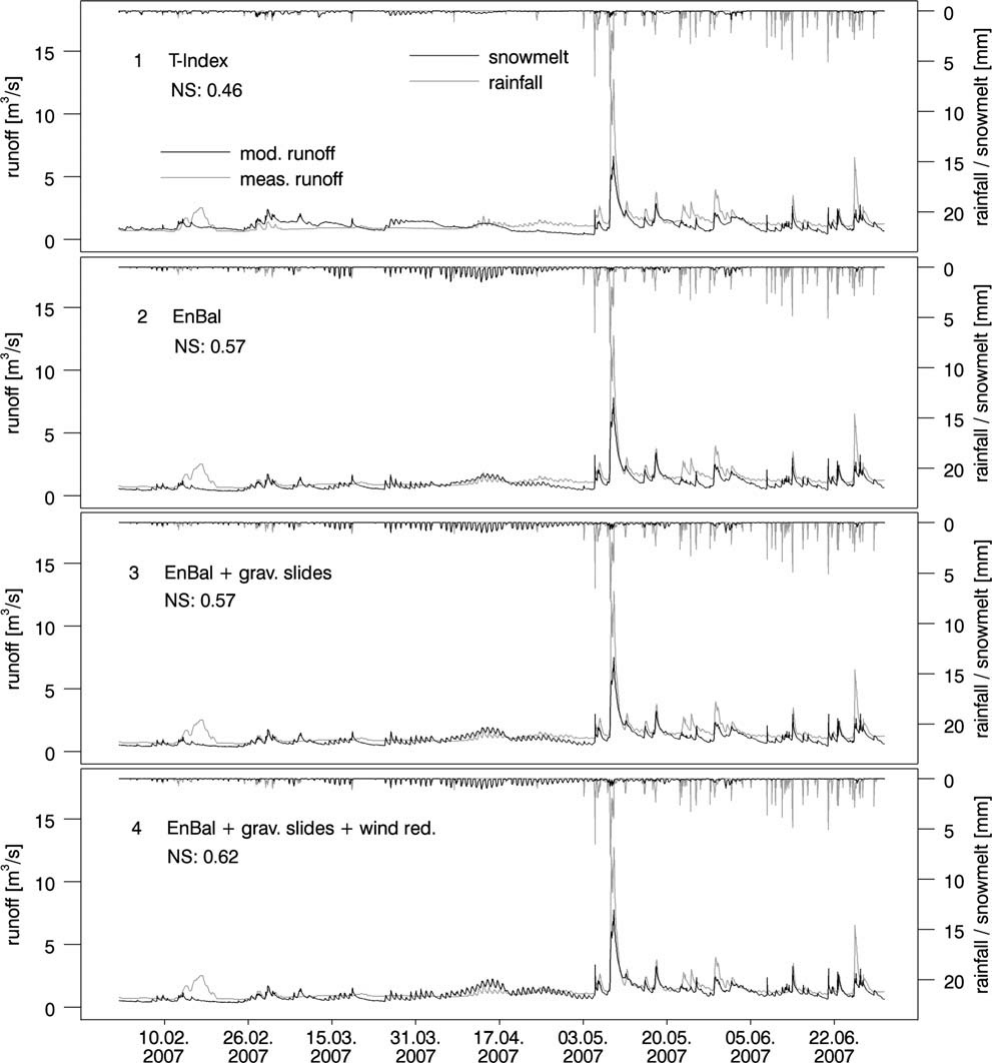
Modeled and measured runoff at gauge Hintersee from February to June 2007 and according to Nash-Sutcliffe coefficients (NS). Increasing snow model complexity from top to bottom: 1, temperature index; 2, energy balance; 3, energy balance and snow slides; 4, energy balance, snow slides, and wind redistribution.

[47] The different modeling approaches produce different snowmelt and runoff dynamics. Figure 10 shows the modeled and measured runoff in m^3^ s^−1^ at the gauge *Hintersee* from February to July 2006 and Figure 11 for the same period in 2007. February–July is the period, when snowmelt plays a significant role in runoff generation in the region. Figures 10 and 11 additionally display modeled rainfall and snowmelt rates in millimeters for the catchment. In this way, snowmelt- and rainfall-triggered runoff events can be distinguished clearly.

[48] The energy balance calculation produces daily fluctuations in the snowmelt rates that agree to the channel stream. This daily cycle of snowmelt and runoff rates could not be reproduced by the temperature index approach (Figure 10, April). As it is run on an hourly basis, there are daily cycles as well, but the magnitudes are much smaller compared to the energy balance calculation. Directly modeling the impact of incoming radiation energy leads to more pronounced melting peaks. These melting peaks promote more clearly to runoff peaks in channel streamflow when routing the water to the gauges.

[49] During the winter, there are various discharge peaks in the simulations that cannot be observed in reality (Figure 10, February, temperature index). These peaks do not occur when using the energy balance approach. This effect can be explained by air temperature exceeding the defined limit for melting conditions in equation (2) for short periods. Melting occurs even if the available energy input is not able to melt snow, because it is used to warm up the cold snowpack. These cases are identified by the energy balance approach where the available energy input is determined explicitly. Consequently, the respective runoff peaks vanish (Figure 10, February). In contrast, a small runoff peak at the beginning of March was not modeled properly by the energy balance method and overestimated by the temperature index calculation. Total melting and runoff rates are highest in the spring months from March to May. Analyzing mean snowmelt totals per month (not shown here) reveals a clear shift of simulated snowmelt from summer, autumn, and early winter months to early and late spring months. At the gauge Hintersee, the Nash-Sutcliffe coefficient for runoff model performance rises from 0.52 to 0.58 during this period when using the energy balance approach (Figure 10). For the whole period modeled, the coefficient increases from 0.57 to 0.62 and the mean coefficient of all gauges from 0.62 to 0.63 (Table 6).

[50] The following melting season 2007 (Figure 11) shows the result of a winter season with different meteorological conditions, generally with less amounts of snow (Figure 2) and the snow cover melting off several times during midwinter over large areas of the catchment. Results are similar to the year 2006, though a runoff peak in February was not captured by the model. This is probably due to a precipitation event in the subcatchment that was not measured by the meteorological stations. The daily fluctuations in snowmelt and runoff are overestimated in some periods by the energy balance calculation during March and April. The Nash-Sutcliffe coefficient increases from 0.46 to 0.57 in the melting season 2007 when using the energy balance calculation instead of the temperature index approach (Figure 11).

[51] Accounting for gravitational snow transport generally shifts runoff from early spring to late spring. This can be explained by snow masses that slide down and accumulate at shaded locations. These are spatially limited areas with very high amounts of incoming snow. They can be identified in Figures 4 and 7 at the base of steep faces where the terrain flattens out. This water storage concentrated on fewer snow-covered cells with large amounts of snow needs time to melt off completely and therefore still produces runoff later in the season. The introduction of gravitational snow transport reduces snowmelt totals in March and April and increases the rates in May, June, and July. The Nash-Sutcliffe performance increases to 0.69 in the melting period 2006 (Figure 10) and to 0.57 in 2007 (Figure 11). Accounting for gravitational snow transport increases runoff model performance regardless of the catchment and time period (Table 6).

[52] Wind-driven redistribution is estimated roughly by a terrain analysis and implemented by correcting the field of snow precipitation. It is not an event-based method or a detailed description of the wind field and snow transport physics, but an assessment of redistribution patterns on a regional scale. Differences in modeled discharge dynamics are a gain of the effects that were introduced by the simulation of gravitational transport. Snow precipitation in winter at steep locations on leeward ridge sides (Figure 5) is highly increased. Consequently, the snow masses are transported laterally down the slopes by the snow slide module. This is a process that can be observed well in reality. The Nash-Sutcliffe coefficient for runoff increases to 0.76 in the melting season 2006 (Figure 10) and to 0.62 in 2007 (Figure 11). Performance does not necessarily increase in every case by introducing wind redistribution. As a consequence of the process described, model performance decreases noticeably when assessing wind redistributions without accounting for gravitational slides (not shown here). In that case, snow masses are transported into steep leeward slopes and not entrained downslope. Therefore, for the simple parameterization of wind-driven snow redistribution used, the simulation of gravitational transport is required to transfer and properly place the wind-enhanced snowfall amounts at the base of steep slopes. This certainly holds for any kind of simulation of lateral wind-driven snow transport in complex, high Alpine terrain where avalanching and sloughing are widely present. Table 6 shows that the simulation of wind-driven redistribution increases Nash-Sutcliffe coefficients for the Alpine headwater catchments. Generally, performance increases depend on catchment characteristics. The more mountainous terrain is present, and the smaller the total size of the catchment is, the larger are the differences. Regarding the entire catchment, the increase in model performance is very small (not evident in Table 6 due to rounding).

## 6. Conclusions

[53] This study aims at investigating effects of complex snow process descriptions on the performance of a hydrological model system in high Alpine terrain. Several high Alpine-specific snow modeling methods were implemented in a distributed hydrological model.

[54] In the complex terrain of the catchment, the integration of the energy balance-based approach to determine snowmelt combined with the simulation of lateral snow transport results in large changes in the spatial distribution of snow cover duration compared to a temperature index method. These large differences lead to relatively small but still remarkable changes in the simulated course of runoff. The diurnal variations in discharge due to snowmelt peaks at daytime are simulated more realistically by introducing the energy balance approach. Furthermore, the seasonal distribution of snowmelt rates and subsequent runoff generation are modeled more accurately. Accounting for gravitational snow transport increases runoff model performance regardless of the catchment and time period. When assessing wind-driven redistribution of snow, model performance for runoff decreases noticeably when gravitational slides are neglected. As a consequence, accounting for gravitational snow transport is a prerequisite for the simulation of wind-driven snow redistribution in this catchment. It is required to transfer and properly place the wind-enhanced snowfall amounts at the base of steep slopes. This might hold for any kind of simulation of lateral wind-driven snow transport in complex, high Alpine terrain. The simulation of wind-driven redistribution increases Nash-Sutcliffe coefficients for discharge at the Alpine headwater catchments, whereas regarding the entire catchment, changes are small.

[55] Some other important processes have not been taken into account so far, e.g., the interaction between snow and vegetation, particularly tree canopy. Interception, sublimation, melt unload, and changes in micrometeorology play a significant role in snow cover development beneath a canopy. *Strasser et al*. [2011] show the influence of tree canopy processes on snow cover development in the region. An interesting modeling enhancement would be the calculation of advective lateral exchange of sensible and latent heat between snow-covered and snow-free ground as that is known to be an important process for snowmelt timing in spring when the snow cover becomes patchy [*Pohl and Marsh*, 2006; *Mott et al*., 2011a].

[56] Future research must focus on measurement techniques to capture temporal dynamics of spatial snow cover distribution. Laser scan measurement—airborne or terrestrial—would be an ideal validation source for modeled snow accumulation and ablation processes [*Prokop et al*., 2008; *Prokop*, 2008; *Dadic et al*., 2010a, 2010b; *Grünewald et al*., 2010; *Mott et al*., 2011b]. Such data would be highly useful for validating this work. Another validation method would be measurements of stable isotopes (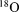 to 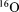 ratio in water) in stored and flowing water compartments allowing to determine the contribution of snowmelt to total measured runoff.
